# Editorial: Exploring immunological tolerance in allergy treatment through AIT

**DOI:** 10.3389/fimmu.2026.1863656

**Published:** 2026-05-26

**Authors:** Caterina Vizzardelli

**Affiliations:** 1 Institute for Pathophysiology and Allergy Research, Medical University of Vienna, Vienna, Austria; 2 Austrian Science Fund (FWF), Vienna, Austria

**Keywords:** allergen immunotherapy, biomarkers, dendritic cells, immune modulation, immune tolerance, nanoparticles, peptide immunotherapy, regulatory T cells

## Introduction

Allergic and autoimmune diseases continue to impose a major global health burden, and despite advances in diagnostics and therapy, the fundamental challenge remains unchanged: identifying safe and durable ways to re-establish immune tolerance. The 16 studies collected in this Research Topic highlight an accelerating shift toward mechanistic, personalized, and multimodal strategies for inducing tolerance. From next-generation allergen immunotherapy (AIT) approaches to metabolic reprogramming, peptide-based tolerance, and engineered cellular therapies, the field is moving toward a more precise and integrated understanding of how tolerance can be restored across distinct disease settings. This editorial synthesizes overarching themes emerging from these contributions and outlines the expanding toolkit available for immune modulation.

## Advances in food allergy immunotherapy: from classical OIT to engineered antigen delivery

Several studies highlight major progress in food allergy immunotherapy. The clinical trial evaluating cashew oral immunotherapy (OIT) demonstrated that 65% of participants achieved both desensitization and sustained unresponsiveness, with immunologic remodeling characterized by a reduction in CRTH2^+^ CD4^+^ T cells and distinct baseline chemokine signatures in high responders. These findings reinforce the relevance of pre-treatment biomarkers in predicting long-term immunologic outcomes.

Complementing these clinical insights, innovations in antigen delivery are reshaping the future of food immunotherapy. Preclinical work using co-encapsulated BLG peptide and CpG nanoparticles shows that spatially coordinated antigen–adjuvant presentation is essential for generating robust tolerogenic responses. Likewise, the development of hypoallergenic recombinant food-based immunotherapies and low-dose OIT strategies reflects a growing emphasis on therapeutic safety without compromising efficacy. Collectively, these studies illustrate a transition toward precision antigen engineering aimed at engaging the immune system more efficiently and with fewer adverse effects.

## Cellular and peptide-based tolerance strategies in autoimmunity and allergy

Restoring immune tolerance remains a central objective across autoimmune disorders. In this issue, peptide-based immunotherapy emerges as a promising strategy. Dose-escalated administration of the TSH-R p37 peptide prevented disease in a Graves’ disease mouse model, with protection dependent on regulatory T-cell (Treg) function and modulation of PD-1^+^ T-cell subsets. Importantly, a weakly immunogenic peptide variant failed to confer similar benefits, underscoring the importance of antigenic fidelity and dose optimization.

Engineered cellular therapies represent another parallel approach. Tolerogenic dendritic cells expressing IL-10, CCR9, and collagen type II successfully reduced disease severity in an arthritis model while promoting Treg expansion and limiting pathogenic T-cell proliferation. These advances position immune-cell engineering as a powerful complement to antigen-specific therapies, with the capability to directly reprogram pathogenic immune networks.

## Adjuvants, immune modulators, and metabolic reprogramming as tolerance amplifiers

A recurring theme across multiple studies is that tolerance induction can be strengthened by manipulating immune signaling pathways or cellular metabolism. Aryl hydrocarbon receptor (AhR) ligands enhanced the efficacy of AIT by diminishing Th2 and Th17 responses, emphasizing the value of environmental sensing pathways in regulating tolerance.

Meanwhile, Notch pathway inhibition synergized with AIT to suppress ILC2-driven type 2 inflammation. This work highlights how modulating innate lymphoid cell plasticity can support long-term immune recalibration.

Perhaps most striking are insights into metabolic reprogramming of antigen-presenting cells. Myeloid dendritic cells stimulated with MPLA-containing AIT products exhibited a glycolytic shift reminiscent of the Warburg effect, which was necessary for Th1-promoting cytokine secretion. Blocking mTOR signaling abrogated these metabolic and immunologic changes, providing a mechanistic link between cellular energy states and tolerance induction. These findings position immune metabolism as an attractive target for directing therapeutic immune responses.

## Innovations in allergen immunotherapy delivery: toward safer, faster, and more accessible care

Delivery route innovations continue to diversify the field of immunotherapy. Pediatric intra-cervical lymphatic immunotherapy (ICLIT) was associated with reduced treatment duration, lower pain perception, and fewer systemic reactions compared to subcutaneous immunotherapy (SCIT). Although SCIT demonstrated better long-term control, the logistical advantages of ICLIT may improve accessibility and adherence, particularly in children.

Recent developments in epicutaneous immunotherapy (EPIT) also demonstrate substantial progress in designing patches and microneedle systems capable of more controlled antigen delivery. By navigating the balance between skin barrier penetration and inflammation minimization, these technologies bring AIT closer to a non-invasive, patient-friendly modality.

Nanoparticle-based adjuvants further enhance delivery strategies. Elastin-like polypeptide (ELP) nanoparticles presenting hypoallergenic Bet v 1 induced strong IgG responses with reduced Th2 skewing—an important step toward replacing alum, whose pro-inflammatory profile has long posed safety concerns. Such advancements contribute to the broader trend toward biomaterial-based immunomodulation, where the therapeutic strategy is guided by design is informed by biophysics as much as immunology.

## Biomarkers and big-data approaches for precision immunotherapy

Multiple contributions emphasize the need for reliable biomarkers to optimize patient selection and monitor treatment progress. Ratios of T follicular helper (Tfh) to T follicular regulatory (Tfr) cells, IgE/IgG4 dynamics, and cytokine signatures such as TARC and IP-10 serve as potential predictors of clinical response. At the population level, regional differences in house dust mite sensitization profiles—such as the prominence of Der p 23 in Ukrainian adults—highlight the importance of tailoring immunotherapy formulations to local allergen exposure patterns.

A bibliometric analysis underscores expanding global research efforts focusing on NF-κB signaling, environmental drivers of sensitization, and personalized therapy. These trends reflect growing recognition that precision immunology must underpin both diagnosis and treatment.

## Conclusions

The collective findings from this Research Topic showcase a rapidly evolving field in which tolerance induction is no longer dependent solely on traditional high-dose immunotherapy. Instead, tolerance is being pursued through engineered antigen delivery, cellular immunotherapy, metabolic modulation, adjuvant innovation, and biomarker-guided personalization. These multidimensional strategies reflect a more sophisticated understanding of immune networks and signal a new era of safe, effective, and durable therapies for allergic and autoimmune diseases.

As the boundaries between clinical immunology, systems biology, and biomaterials science continue to blur, the field is poised to achieve increasingly precise and long-lasting immune tolerance.

